# Hierarchical cloud architecture for identifying the bite of “Egyptian cobra” based on deep learning and quantum particle swarm optimization

**DOI:** 10.1038/s41598-023-32414-w

**Published:** 2023-03-31

**Authors:** Ahmed Hassan, Mohamed Elhoseny, Mohammed Kayed

**Affiliations:** 1 Faculty of Science, Beni-Suef University, Beni-Suef, 62511 Egypt; 2 Faculty of Computers and Information, Mansoura University, Mansoura, 35516 Egypt; 3 Faculty of Computers and Artificial Intelligence, Beni-Suef University, Beni-Suef, 62511 Egypt

**Keywords:** Drug discovery, Zoology

## Abstract

One of the most dangerous snake species is the “Egyptian cobra” which can kill a man in only 15 min. This paper uses deep learning techniques to identify the Egyptian cobra bite in an accurate manner based on an image of the marks of the bites. We build a dataset consisting of 500 images of cobra bites marks and 600 images of marks of other species of snakes that exist in Egypt. We utilize techniques such as multi-task learning, transfer learning and data augmentation to boost the generalization and accuracy of our model. We have achieved 90.9% of accuracy. We must keep the availability and accuracy of our model as much as possible. So, we utilize cloud and edge computing techniques to enhance the availability of our model. We have achieved 90.9% of accuracy, which is considered as an efficient result, not 100%, so it is normal for the system to perform sometimes wrong classifications. So, we suggest to re-train our model with the wrong predictions, whereas the edge computing units, where the classifier task is positioned, resend the wrong predictions to the cloud model, where the training process occurs, to retrain the model. This enhances the accuracy to the best level after a small period and increases the dataset size. We use the quantum particle swarm optimization technique to determine the optimal required number of edge nodes.

## Introduction

According to the world health organization (WHO), there are 5 million persons are exposed to snake bites annually. This leads to occurring 2.5 million cases of poisoning, 137.880 deaths and also 300.000 cases of amputation and paralysis per year. So, snakes are among the most three dangerous animals to humans. Out of 3848 identified species of snakes, around 800 species are venomous, among which only about 50 species are fatal to humans. The “Egyptian cobra” is of the most poisonous snakes in my country “Egypt” and its venom is able to kill a man in only 15 min, and an elephant in 3 h^1,2^. Egyptian cobras prefer to stay near the human houses to hunt domestic fowl like chickens. This increases its danger to the Egyptian people. Generally, one of the quickest required measures after a snake bite is to identify the species of the attacking snake to take the right antitoxin. In real situations, doctors mostly give polyvalent anti-venom to the snake bite victim without considering which snake species has bitten the victim. Even if the patient observed some visual features of the snake, the medical person is not expert in the snakes species to determine the correct species of the attacking snake from the description taken from the victim. Also, the victim in this situation is not aware enough to focus on snake features. In the case of the polyvalent anti-venom is given to the victim, polyvalent anti-venom includes antibodies against more than one snake species. So, polyvalent anti-venom may neutralize the venom of a single snake bite and the remaining non-neutralized part of the polyvalent anti-venom causes further risk to the human health^3^. So proper identification of the snake is very important for the proper medical treatment to save the life of the snake bite victims^4^.

Computer vision technique has developed quickly in the area of automated object classification and recognition^5–8^. Computer scientists use different machine learning techniques for image classification. Image classification, based on machine learning, contains two phases: feature extraction and classification. During training, the pre-defined classes (labels) can be conceived of the available dataset that extracts the characteristic features from each image class and forms a special description for each specific class. Using machine learning for the recognition of plants and animals’ images is growing quickly. Recently, efforts have been made for machine learning based snake classification^9–11^. Whereas the bite marks is one of the common factors to identify the species of the snakes^12^, we train a deep learning model on a big dataset of 2 classes: images for bite marks of Egyptian Cobra and the second class contains images for bite marks of other snake species. After training the model on this dataset, it is able to classify a snake bite marks whether it is an Egyptian cobra species or not. So, in this paper, we study the efficiency of using bite marks as a training material for the model to perform efficient snake species identification.

We utilize cloud and edge computing techniques to enhance the availability of our model and to take the decision in a very low time using edge computing technique and to enhance the accuracy, in a more manner, gradually. Deep learning relies on the availability of a high computing environment with large storage, needed for the data required to train these models^13^. Cloud computing, where a huge amount of processing capability and storage is available, is the preferred computing platform for operating deep learning models^14^. However, the low-latency and availability requirements of some deep learning applications are challenging this cloud (centralized) computing model to present the required quality of service. The availability of our classifier is vital in our application because if the centralized cloud computing is not available, the victim will die in only 15 min. Many deep learning applications have low latency requirement. For example, our application requires a very quick decision because the “Egyptian Cobra”, as we said before, is able to kill a man in only 15 min. So, our deep learning application requires closer computing node. These challenges lead to the using of the edge computing paradigm^15^ because it provides a distributed closer computing node. So, in each state, a number of edge nodes, where our classifier application is positioned, are deployed and connected to the cloud computing node where the training process is done. Our model achieves 90% accuracy which is considered as a very efficient result, but there is 10% error rate which is very normal until with the human doctors. However, we want to reach the best possible accuracy along time. So, we also propose a novel re-training approach where the cloud architecture is later responsible also for storing the updated dataset with any case that is predicted incorrectly by the model (during model usage) to retrain the model with this updated dataset to reach the best accuracy along time and generate a more accurate version of the model. So, the cloud is represented as central point to receive the updates of the dataset from all edge nodes to enhance model accuracy.

Here, we can list the contributions of our proposed model as shown below:

1. To the best of our knowledge, this is the first attempt to recognize the species of the attacking snake based on the bite marks which is more practical for this situation than previous works that need an image for the snake which may be not available.
2. We use both edge and cloud computing paradigms to enhance the availability of the model and reduce the decision time. Also, we used quantum particle swarm optimization for determining the optimal number of deployed edge nodes considering: cost, delay and ratio of real dangerous cases because of snake bites in this area.
3. Differently from previous models that consider their models as final versions, we suggest using our model and training it in a continuous manner, after the basic training process, to reach the best accuracy level by updating the dataset and retraining the model with its wrong prediction decisions to generate more accurate version along time.

This paper is organized as follow: “Related works” section discusses the existing literature; proposed classification model is discussed in the “Methodology” section; performance analyses are discussed in the “Performance evaluation” section; the “Conclusion” section concludes the paper.

## Related works

**Figure 1:**
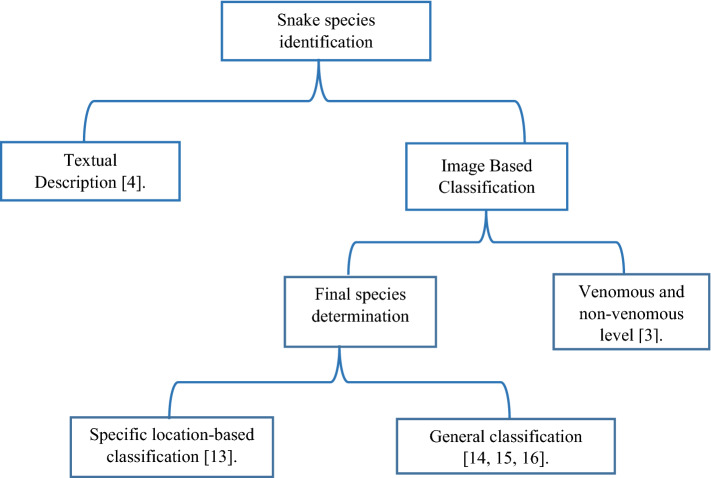
Taxonomy of deep learning based snake species identification.

We made an efficient taxonomy of the previous models that were used to make snakes species identification to help millions of people that are exposed to the snakes’ bites, as shown in Fig. 1. Firstly, we categorize the paper based on the input of the user, whereas some models take a textual representation for the attacking snake from the user and other take image and make image classification. Some papers then stop in the classification process to the level that determining only whether the snake is venomous or not, but the other category continues to determine finally the name of the species of the snake. Also, some paper classify the snakes only in a specific location only, but the other classifies generally. Generally, intelligent machine learning techniques, including deep learning algorithms, are used to handle the danger of the snakes which are considered as one of the highest three reasons of death for humans^16^. In a previous study, images of 22 species of snakes that exist in Malaysia were collected into a dataset, namely the Snakes of Perlis Corpus. After that, intelligent techniques, such as k-nearest neighbors, nearest neighbors (k-NN) and backpropagation neural network, are used to automatically recognize a snake species based on an input image with 87% accuracy^17^. The snake species could be manually recognized based on some visual features such as head shape, body texture, skin color, and eye form, which are not easy for non-expert people. In another previous work, a convolutional neural network (CNN) is used to develop classification of snake species based on an input image of the snake. Three CNN is trained using a dataset of 415 images of snakes from five popular venomous snake species in Indonesia.

Another previous study used AI on collected images of the snakes to generate an AI platform that is able to identify the species of a snake based on a user’s uploaded image and informs users with information about the natural history, distribution, conservation, and etymology of this snake species^10^. A Deep neural network has been suggested to classify snakes into two classes: venomous and non-venomous, with high accuracy of 91.30%^10^. A dataset consists of 1766 snake pictures is utilized to develop seven Neural networks. The dataset has been increased by utilizing various techniques. Also, the transfer learning technique is used to enhance the identification accuracy even more. Another previous study proposes a model that recognizes the snake species through visual description taken from the victim about the observable visual features such as head shape, skin color, etc. based on training and classification techniques in Machine learning^11^. We can note that the above papers depend in their classification process on the visual characteristic of the snake, whether using an image or description from the victim. Another previous work involves algorithms based on Image Processing, Convolution Neural Networks and Deep Learning to achieve the identification of snakes species. In most cases, extracting features and utilizing them for classification. Firstly, “GrabCut” algorithm is used with the images to extract the foreground of the images and eliminate any useless information^18^. After that, the system is trained and combined with a Pre-trained model to predict class labels. In a previous work, snake species are identified efficiently, whereas instead of considering predictions with the recent model, an ensemble model is utilized^19^. So, the last 5 epochs of the used models were considered. After that, the predictions resulted from each model are averaged, and then the average of the predictions resulted from all models is taken. This increases the accuracy of the model manifold.

The “SnakeCLEF 2021 challenge”, presents a large dataset including images and their recording location for 772 snake species. In a previous work^20^, deep learning- models were trained to recognize snakes species. Also, the prior probabilities of the location data were multiplied by the model predictions. An ensemble of the used deep learning techniques achieved the best results, which was 82.88% of accuracy. A previous work suggests an object detection and blurring system based on deep learning for the recognition of snake images since while some people do not care about the emergence of the snake images on the visual platforms, there is a portion of them who are uncomfortable of watching snakes images^21^. So, with this system, the snake objects from the images are detected using YOLOv4 trained model and blurred with OpenCV. The detection f1 score of the selected model is 92% on the test set.

### Evaluating the discussed deep learning based snakes species identification

The above techniques are not practical in a real scenario because after a victim is bitten by the snake, he cannot save its features, and the victim has not the time to take a picture. So, our model is more efficient because it depends on the bite marks that can be checked any time after the bite. Also, bite marks are considered as one of the factors that characterize each snake species^22^. Careful observation of the snake’s bite site can be very useful, together with the search for signs and symptoms of local and systemic envenomation, to provide the best possible diagnosis. Regarding the category of the papers that determine only the attacking snakes are venomous or non-venomous, determining whether the bite was by a venomous or non-venomous snake may prevent useless administration of antivenin, which sometimes can, by itself, cause untoward effects, including life-threatening hypersensitivity reactions. We can also note that the papers of the category location-based classification, that identify the snakes species that exist only in a specific location, achieve more accuracy compared to the papers that make general classifications of all snakes species, because there are a very big number of snakes species (3848 known species). So, having a dataset that contains a significant number of images of each species from all snakes species is a very complex task.

Whereas the Egyptian Cobra is able to kill a man in only 15 min, we must keep the availability and accuracy of our model as much as possible. So, we utilize cloud and edge computing techniques to enhance the availability of our model and to take the decision in a very low time using edge computing technique. We present a general comparison between the cloud and edge computing paradigms, as shown in Table 1. We present a comparison among the reviewed papers and our proposal, as shown in Table 2.

**Table 1:** A comparison between the cloud and edge computing paradigms.

Dimension	Cloud computing	Edge computing
Delay	More: proximity of the services to the user is low.	Less: closer to the user location
Computing power	more	Less
Availability	Low: only one centralized computing node. If this node is exposed to any corruption, the system breakdowns	High: some distributed node
Security	Transferring data to the cloud using public shared network reduces the security and privacy	Edge paradigm reduces data-sensitive transfer which makes the data more private and secure
management	Centralized services are easy to be managed by the user	Distributed services are difficult to be managed by the user

**Table 2:** Comparison among the reviewed papers and our proposal.

References	Data set	Classification technique	Evaluation
^17^	Images of 22 snake species	Clustering	Image of the attacking snake is required which is not practical
^10^	Snake images	Deep learning	The model output is not enough and wastes time when dealing with critical cases
^11^	Snake images	Machine learning	The final model requires visual description from victim to predict the species of the snake. However, this is not convenient because the victim is not in his complete awareness
^19^	Snake image	Ensemble learning	The user inputs an image for the attacking snake to get the classification, however this image may not appear clear descriptions for the snake because it may exist in a harsh environment in a place which it is not easy to reach
^21^	Images contain snakes	Deep learning	In most of these images the part of the snake does not appear in the image or the snake coiled around itself. So, some features of the snake do not appear in these images and the model do not train on them
^27^	Images for snakes with their geographical information	Deep learning	Depending on the geographical distribution- information in the recognition process is not sufficient, because in countries like India there exist 400 snake species. So, recognizing a snake species among them stills a difficult task
^28^	Images for 198 venomous snake species and 574 non-venomous snake species	A recent neural network architecture	Basic variance between the practical situations and the samples that the model trained on them
Proposed model	Images of snake bite marks	Ensemble learning	This is the first attempt for a model to identify snake species based on bite marks. This is more practical and accurate, as shown in Table 6

## Methodology

Whereas snakes species identifications is the first process to treat a snake bite victim. Deep learning techniques have been used in many healthcare tasks, and achieve great success. This model uses deep learning techniques to identify the Egyptian cobra bite in an accurate manner based on an image of the marks of the bites. We build a dataset consisting of 500 images of cobra bites marks and 600 images of marks of other species of snakes that exist in Egypt. We utilize techniques such as transfer learning and data augmentation to boost the generalization and accuracy of our model.

### Dataset collecting

**Figure 2:**
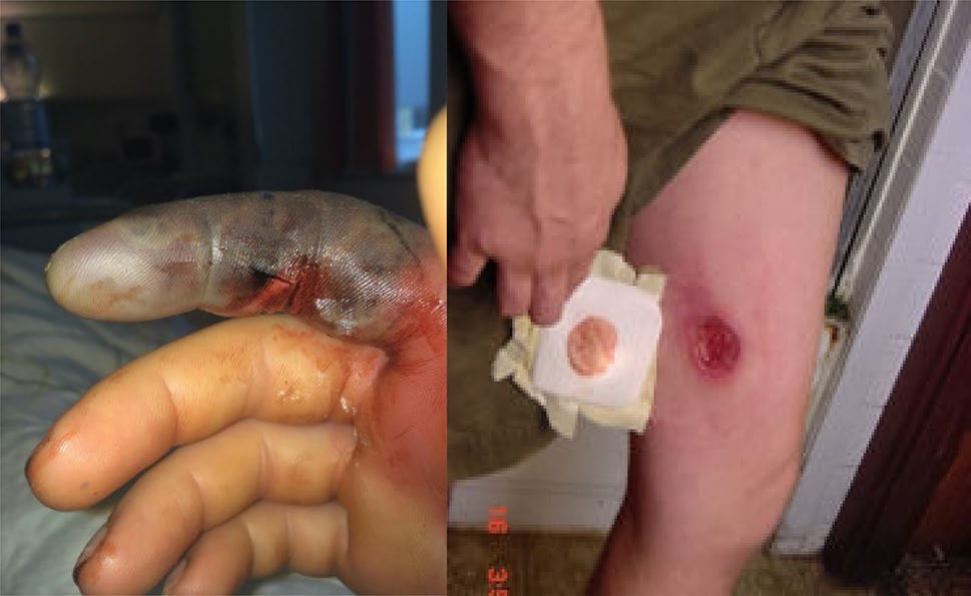
Bite marks of Egyptian cobra with two victims^19^.

We have faced a big challenge in that we have not found an available dataset that is convenient to our target. Also, the pictures of bite marks, that we need to train our model, are not generally available in a significant manner online because they must be shared after the agreement of the patient. So, we have to search online deeply to find pictures of bite marks of Egyptian cobra’s victims (this increases the difficulty of our mission). After searching in the zoology and medical scientific papers and online medical websites^19,23–25^, we found 847 pictures of bite marks of the Egyptian cobra and 1005 images of other snakes bites marks, as shown in Fig. 2. This is not a very bad number considering the above difficulties, but we have to handle this challenge to get the best possible accuracy. We use data augmentation and transfer learning to handle this challenge. Whereas the size of the generated images in the data set is not the same and the sizes of images are different. So, we have changed the size of all the bite marks images into the same size of 1000 × 1000 pixels. For this, RGB reordering has been achieved and the input to the proposed model is presented as 1000 × 1000 × 3 image. The victims in this dataset are from different nationalities and ages, 73% of them are male and 27% are female, 54% of them are young and 46%of them are old people, part of them suffer from chronic diseases.

### Dataset pre-processing

#### Dataset enhancement

**Figure 3:**
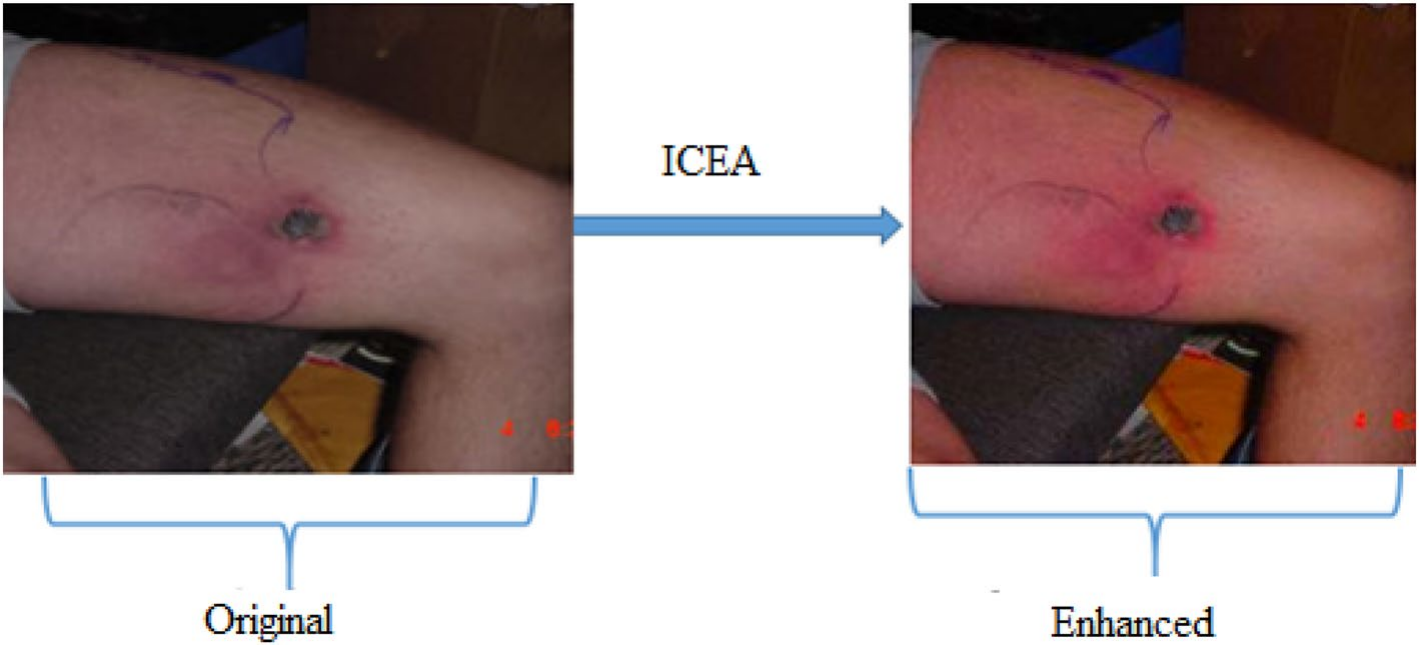
Enhancement process for the dataset samples.

Compared with other image data in many research fields, most of the images of bitten persons by snakes are taken in poor countries, such as Egypt, Africa and India. So, the quality of these images is poor and the images backgrounds are usually complex. Image preprocessing is a very important process to remove damaged images, get rid of the effect of noise. During the process of generating the enhanced data set, contrast enhancement was applied on each image existed in the original dataset, using the image contrast enhancement algorithm (ICEA), as shown in Fig. 3. By this way, the noise in the original data set was eliminated and the best contrast was obtained. The ICEA is considered as one of the best image processing techniques used as a solution for the contrast enhancement issue. We used a new and efficient algorithm, for generating the enhanced dataset, which was proposed by Ying et al.^22^.

$$Rc = \mathop \sum \limits_{i = 1}^{N} W_{i } P_{i}^{c}$$

$$P_{i} = \, g\left( {P, \, k_{i} } \right)$$

where R represents the result of enhancement, c represents the index of the three-color channels, and N is the number of images, W_i_ is the i-th image’s weight map, and P_i_ is the i-th image in the exposure set. Also, g is the Brightness Transform Function, and k_i_ is the exposure ratio.

#### Dataset augmentation

Enhancing the generalization feature of the deep learning models is one of the most important challenges. Generalizability points to the performance of a model when dealing with data that it has not seen before (testing data) after training on previously seen data (training data). This needs a very large dataset to learn many features. However, there is sometimes a limited dataset. So, we have to handle this challenge to enhance the accuracy of the model. So, we the use data augmentation technique (DA). DA is a mechanism that can be utilized to artificially increase the size of a training data by generating modified data from the existing one. DA is proven that it achieves the best accuracy compared to other techniques that handle the limited dataset challenge. In this paper, flipping the image and zooming-in (zooming the image randomly by a certain degree) augmentation techniques are used to increase the dataset size, as shown in Table 3.

**Table 3:** Values of augmentation parameters.

Augmentation technique	Degree
Flipping	90
Zooming	0.5–1.0

### Transfer learning

Transfer learning is an efficient method in which we reuse a pre-trained deep learning model as a starting point to a model of a new task. In other word, a model trained previously on a task is reused on a second, related task to perform rapid progress while modeling the second task. By implementing the transfer learning on a new task, the model can achieve higher performance than training the model with a small amount of data. In this model, 3 popular pre-trained models were used to classify bite marks of the Egyptian Cobra from other species of snakes in Egypt: (1) VGG19, (2) VGG-16, (3) ResNet-101. VGG-19 consists of 19 layers, including 5 convolutional blocks and 3 fully-connected layers. Compared to VGG-16 model, VGG-19 is a deeper CNN architecture. ResNet-101 is a version of “Resnet” CNN. It is 101 layers depth with 33 residual blocks.

In our model, the convolution layer has a big importance because it does the feature extraction task. This layer utilizes the convolution operation which is alternative for the general matrix multiplication and is the basic building block of CNN. Also, the parameters of the convolution layer consist of a set of learnable filters, also called kernels. The basic task of the convolutional layer is to recognize the features in the regions of the input image and to generate a feature map.

This process depends on the technique of wandering a specific filter on the input image. The size of the filter can be 3 × 3, 5 × 5 or 7 × 7 pixels. The input to the next layer is created with the filter performed to the image. Activation maps happens as a result from this convolution process. Activation maps have local distinctive features.

1 $${Y}_{i}^{(l)}=f \left({B}_{i}^{(l)}+ \sum_{j=1}^{{m}_{i}^{l-1}}\left({K}_{i,j}^{l}+ {Y}_{j}^{(l-1)}\right)\right)$$

Here, each convolution layer has a filter (m_1_). The output of Layer l contains ${m}_{1}^{(l)}$ feature maps in ${Y}_{i}^{(l)}$, ${m}_{2}^{l}* {m}_{3}^{l}$ dimension. The i. feature map shown with ${Y}_{i}^{l}$ is calculated according to Eq. (1), ${B}_{i}^{(l)}$ is the deviation matrix, and ${K}_{i,j}^{l}$ is the filter dimension.

Pooling layers reduce the image dimension gradually so the number of the parameters and calculation complexity of the model are reduced. The pooling layer has two parameters, (l) the dimension of ${F}^{(l)}$ filter and ${S}^{(l)}$ step. The input of this layer is the data in the dimension ${m}_{1}^{l-1}* {m}_{2}^{l-1}* {m}_{3}^{l-1}$, provides ${m}_{1}^{l}* {m}_{2}^{l}* {m}_{3}^{l}$ output volume. The operation of the pooling layer is described in Eqs. (2), (3) and (4).

2 $${m}_{1}^{l}= {m}_{1}^{l-1}$$

3 $${m}_{2}^{l}= {(m}_{2}^{l-1}-{F}^{l}) / {S}^{\left(l\right)}+1$$

4 $${m}_{3}^{l}= {(m}_{3}^{l-1}-{F}^{l}) / {S}^{\left(l\right)}+1$$

In the pooling operation, a v vector is decreased to a single scaler f (v) with pooling process f. There are two pooling types: average pooling, and max pooling. Max pooling is utilized here. FC layers turn the feature maps output of (1) final convolution or pooling layer into a unidimensional vector, (2) bind to one or more dense layers (3) update the weights, (4) and give the predicted of the final classification.

### Proposed cloud architecture

We make use of cloud and edge computing techniques to boost the availability of our system and to decide at a very low time using edge computing technique. Whereas basic cloud unit is responsible for the training process for the model, whereas the storage and the computing capabilities of the cloud unit is convenient to perform this task in an efficient manner.

#### Edge computing structure

Edge computing is a technology utilized to enhance the efficiency of cloud computing architecture. In edge computing, specific parts of the system or services are transferred from the cloud unit to another close logical endpoint (the “edge”). The responsibility of the edge unit is to present fast services to the remote areas being served. When an application is started on the edge, it can provide a faster response to meet the requirements of real-time identification of Egyptian Cobra bite in remote areas, whereas Egyptian Cobra is able to kill a man in only 15 min. This requires fast identification of the bite. We suggest that in each state in Egypt, there is a number of edge computing units that contain the classifier application. This reduces the required time for the classification process because the classifier is closer to the user than to the cloud unit.

#### Novel re-training task in the cloud architecture

We have achieved 90.9% of accuracy, which is considered as an efficient result, not 100%, so it is normal for the system to perform sometimes wrong classifications. So, we suggest to re-train our model with the wrong predictions, whereas the edge computing units, where the classifier task is positioned, resend the wrong predictions to the cloud model, where the training process occurs, to retrain the model. This enhances the accuracy to the best level after a small period and increases the dataset size.

#### Determining the optimal number of edge nodes using quantum PSO algorithm

**Figure 4:**
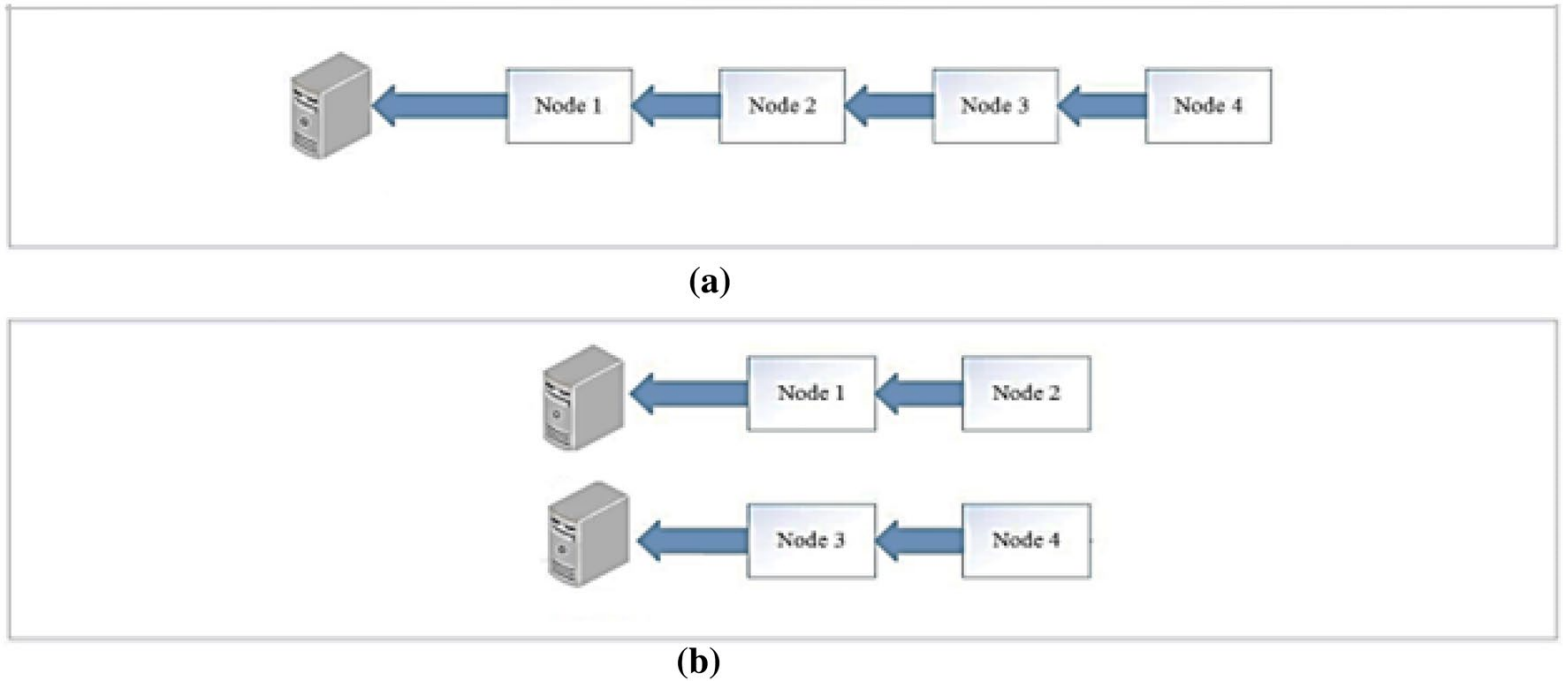
The effect of edge nodes’ number on delay.

In each state, a number of edge nodes are deployed to present the classifier service to the users in this state. The optimal number of deployed edge nodes has to be determined based on some factors:

1. Delay reduction: the Egyptian Cobra is able to kill a man in only 15 min, so the classification should be determined as quick as possible. The number of edge nodes affects the delay, as shown in Fig. 4, where in scenario a, if node 4 wants to send a data packet, it will wait for 4 hops delay. However, in scenario b, node 4 waits only 2 hops to reach an edge node. So, we can say that delay magnitude depends on the number of edge nodes, as shown in Eq. (5).
  5 $$D \alpha \frac{1}{k};$$
  where D is the delay magnitude and k is the number of edge nodes.
2. The bigger number of snake bites in a state is not necessarily a requirement for the more edge nodes existence. However, we suggest that these snake bites may be from non-venomous snakes. So, we consider the ratio of the number of deaths besides the number of blind cases or danger cases to the number of all snake bites in a state, as shown in Eq. (6). So, this ratio has to be close to the ratio of the number of edge nodes in this state to the number of edge nodes in all states. This leads to that each state contains the number of edge nodes that is convenient to its danger ratio.
  6 $$\frac{k}{K} - \frac{p + d}{n} \cong 0;$$
  where k is the candidate number of edge nodes, K is the number of all edge nodes, p number of blind cases or any dangerous cases resulted from snakes bites in this states, d is the number of death cases resulted from snakes and n is the number of all snakes bites in this state.
3. Cost (C): we must consider the cost of buying these edge nodes in each state to achieve the least cost.

So, we have a maximization objective function to find the optimal number of edge nodes that achieves the best profit, as shown in Eq. (7).

7 $$Profit = \frac{1}{k} - \left( {\frac{k}{K} - \frac{p + d}{n}} \right) - C$$

We use particle swarm optimization (PSO) and quantum particle swarm optimization to solve the above optimization problem. So, the output of this process is an integer number which represent the optimal number of edge nodes to an area considering cost, delay and real danger range in this area.

### Particle swarm optimization

PSO is an optimization algorithm inspired from birds flock when searching for the food. In PSO, the candidate solution is called a particle. Each particle has two variables: position and velocity, using them the particle moves through the search space towards the optimal solution^16^. PSO is an iterative technique. In each iteration, it checks the particle position (value) using the fitness function, and saves the particle’s local optimal value and the global optimal value for all particles. Based on the local and global optimal values, the particle updates the velocity that is utilized to calculate a new location for the particle (new candidate solution). The location and velocity of the particle are calculated using Eqs. (8) and (9), respectively. PSO has basic advantages : efficient solutions, ease of implementations, and computational and memory efficiency use.

8 $${v}_{i}\left(t\right)=\omega {v}_{i}\left(t-1\right)+{{c}_{1}{\varphi }_{1}({P}_{i}-x}_{i}(t-1))+{{c}_{2}{\varphi }_{2}({Pg}-x}_{i}(t-1))$$

9 $${x}_{i}\left(t\right)={x}_{i}\left(t-1\right)+{v}_{i}(t)$$

where ${v}_{i}(t)$ is the velocity of particle $i$ at round $t$, ${x}_{i}(t)$ is the position of particle $i$ at round $t$, *w* is the inertia factor, ${c}_{1}$ and ${c}_{2}$ are two positive constants, ${P}_{i}$ is the local best of particle i, $Pg$ is the global best of all particles, ${\varphi }_{1}$ and ${\varphi }_{2}$ are two random number between [0, 1].

### Quantum PSO (QPSO)

The basic disadvantage of the traditional PSO algorithm is that it does not guarantee to reach the global optimal solution (Bergh 2001). Because of the small velocity of the particle, the particle is not able to cover the search space in an efficient manner. QPSO was proposed to handle this disadvantage of PSO. In quantum physics, the position and velocity of a particle could not be calculated at the same time. Therefore, the motion of a particle is described by a wave function denoted as *ψ (x)* that points to the probability of a particle to exist in a specific location (x) at a specific time. Based on Monte Carlo methodology, the motion state of a particle is described according to Eq. (10).

10 $$x_{i} (t + 1) = \left\{ {\begin{array}{*{20}l} {PP_{i} + \alpha \left| {m_{best} - x_{i} \left( t \right)} \right|*\ln \left( {1/u} \right) ,} \hfill & {if \;b \ge 0.5} \hfill \\ {P_{i} - \alpha \left| {m_{best} - x_{i} \left( t \right)} \right|*\ln \left( {1/u} \right),} \hfill & { if\; b < 0.5} \hfill \\ \end{array} } \right.$$

where *PP*_*i*_ is the local attractor of particle $i$ that can be calculated based on Eq. (11),* m*_*best*_ is the mean personal best of all particles which can be determined according to Eq. (12), $\alpha$ (Contraction–Expansion coefficient) can be tuned to determine the convergence speed,, *u and b are* random numbers between [0, 1] and ${P}_{i}$ is the local best of particle i.

11 $$PPi \, = \phi P_{i} + (1 - \phi )Pg$$

12 $$mbest = \left( {\frac{1}{s}\mathop \sum \limits_{i = 1}^{s} PP_{i1} , \frac{1}{s}\mathop \sum \limits_{i = 1}^{s} PP_{i2} , \frac{1}{s}\mathop \sum \limits_{i = 1}^{s} PP_{i3} , \ldots \ldots , \frac{1}{s}\mathop \sum \limits_{i = 1}^{s} PP_{iD} } \right)$$

where ${Pg}$ is the global best solution, *φ* is a random number between [0, 1],s is the swarm size and *D* is the number of dimensions in each particle. Compared to the traditional PSO, QPSO enhances greatly the global search ability and searches in wide space because of the exponential distribution of particle position, as shown in Eq. (10).

## Performance evaluation

The performance of our model to classify marks of snakes bites specifically grouped into two classes, Egyptian cobra and non-Egyptian Cobra, will be covered in this section. We executed too many experiments to adjust the optimal values for the training parameters to get the best performance, as shown in Table 4.

**Table 4:** Training and QPSO parameters.

Parameter	Value
Epochs	100
Learning rate	0.001
Epsilon	1e^−10^
Batch size	64
$\alpha$(Contraction–Expansion coefficient of QPSO)	0.7

### Confusion matrix

Confusion matrix is utilized to evaluate the performance of deep learning models, whereas Confusion matrix is a matrix in which target predictions and actual values are compared to evaluate the classification performance used in deep learning algorithms. The confusion matrix consists of four factors: False Positive (FP), True Positive (TP), True Negative (TN), and finally False Negative (FN), as shown in Table 5. The rows of the confusion matrix contain the number ‘Real class values’, and the columns of the confusion matrix are the number of ‘Predicted class values. This checks the performance of our model. According to the confusion matrix of the validation data set, our model has gotten the sensitivity of 96.7% and specificity of 71.5 %. Whereas Sensitivity indicates the number of True Positives (TP) or the number of times in which the model forecasts that they are marks of Egyptian cobra bite and they are really marks of Egyptian cobra bite So, out of the 220 snake bite in the test data set we were accurately predicted these bites are from Egyptian Cobra in 170 of them presenting 96.7% probability. This shows that we identify marks of Egyptian cobra bites with only 3.3% error which is a very small probability. This reflects the efficiency of our model to identify the Egyptian cobra bites from other snake bites. Specificity points to the number of True Negatives (TN) or the number of times in which the model forecasts they are not marks of Egyptian Cobra bite and they are actually not marks of Egyptian Cobra bite. We has achieved 71.4%. By calculating the accuracy of our model based on the confusion matrix we have achieved an overall accuracy of 90.9 % which indicates that our model is very accurate compared to the accuracy of other snake species- identification models, as shown in Table 6. This is because the training material of our model (bite marks images) presents efficient features for our model to help it efficiently classify the bite marks cases because the structural characteristics and functional properties of the specific venom components from different snake species are different^26^ which makes the classification task performed based on clear parameters.

**Table 5:** Confusion matrix of our model.

Actual	Predicted	
	Cobra	other
Cobra	TP = 170	FN = 8
other	FP = 12	TN = 30

**Table 6:** Practical comparison among our model and other snakes species identification models.

References	Accuracy (%)	Prediction time (ms)
^9^	75	5.186
^29^	82	6.844
^17^	87.93	5.554
Proposed model	90.9	4.262

### Accuracy

**Figure 5:**
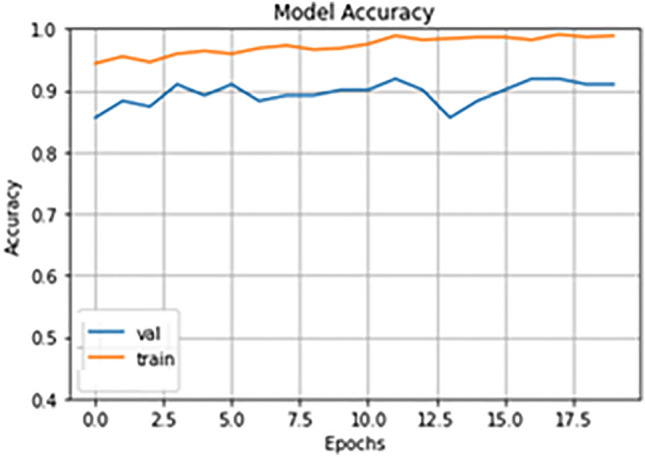
Training and validation accuracy of our model.

We trained our model for 20 epochs and also the learning rate was 0.0001. The validation accuracy lies around 85.5–91.8%, as shown in Fig. 5. The training accuracy lies around 94.3–98.9%, as shown in Fig. 3. We can note that our model achieves not only efficient training accuracy, but also it achieves efficient validation accuracy. This means that our model achieves the generalization feature which is one of the most important requirements of any deep learning model. Our model outperforms the efficient models that make deep learning based snakes species identification, as shown in Table 6, because other approaches trained on classes of snakes images, and this is not efficient because in most of these images the part of the snake does not appear in the image or the snake coiled around itself. So, some features of the snake do not appear in these images and the model do not train on them.

### Area under the curve (AUC)

**Figure 6:**
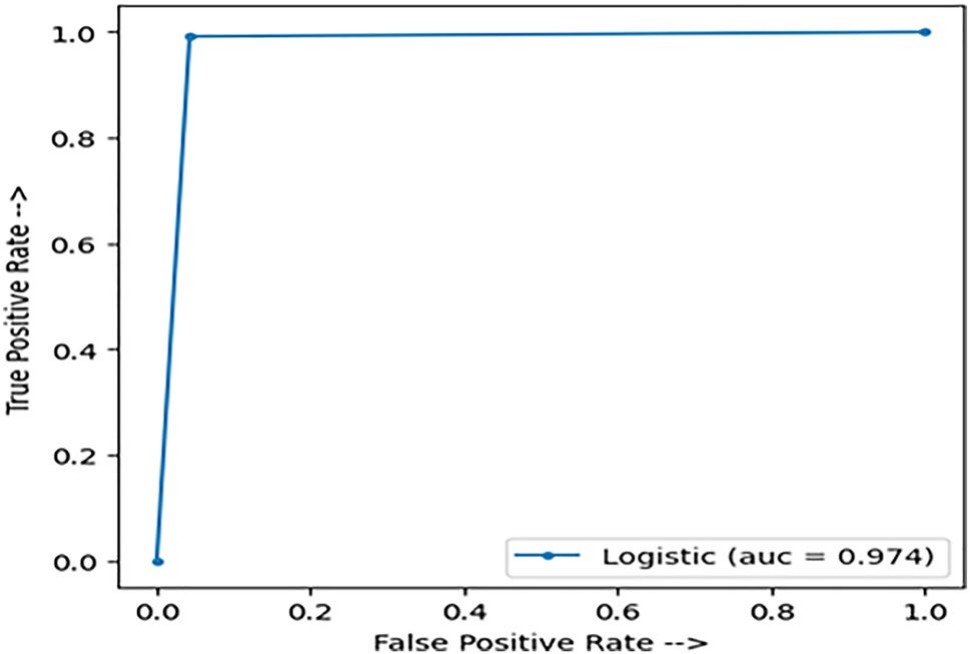
AUC of our model.

AUC points to the classifier’s capability of classifying different classes. The higher AUC value the classifier has, the more efficient in distinguishing between classes. Whereas an AUC close to 0.50 shows that the classifier makes guesses and has no separation amplitude. A low value in AUC shows that the model is predicting classes in the opposite. We have got AUC of 97.4% which is considered as a strong indicator for the efficiency of our model, as shown in Figure 6.

### Loss

**Figure 7:**
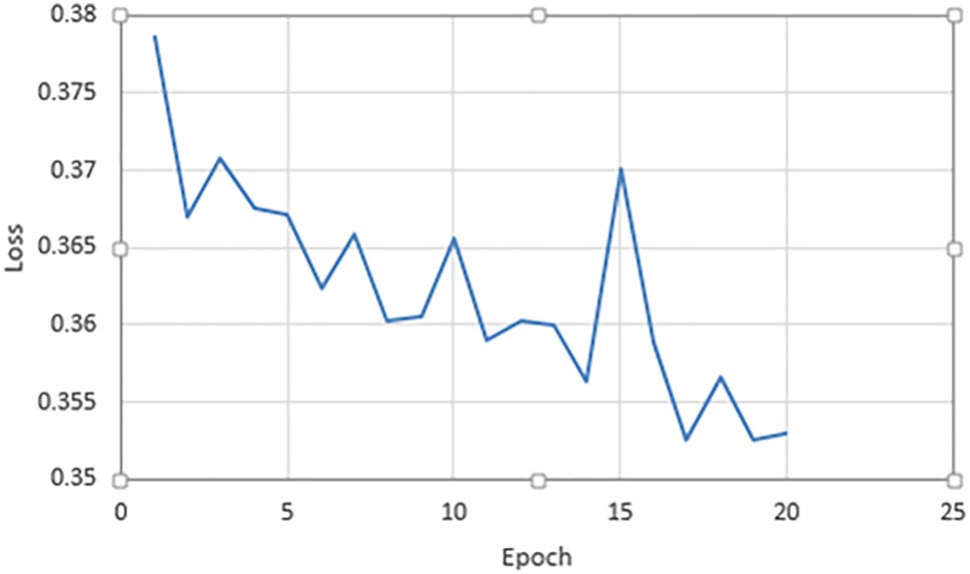
Validation loss of our model for Resnet101 architecture.

**Figure 8:**
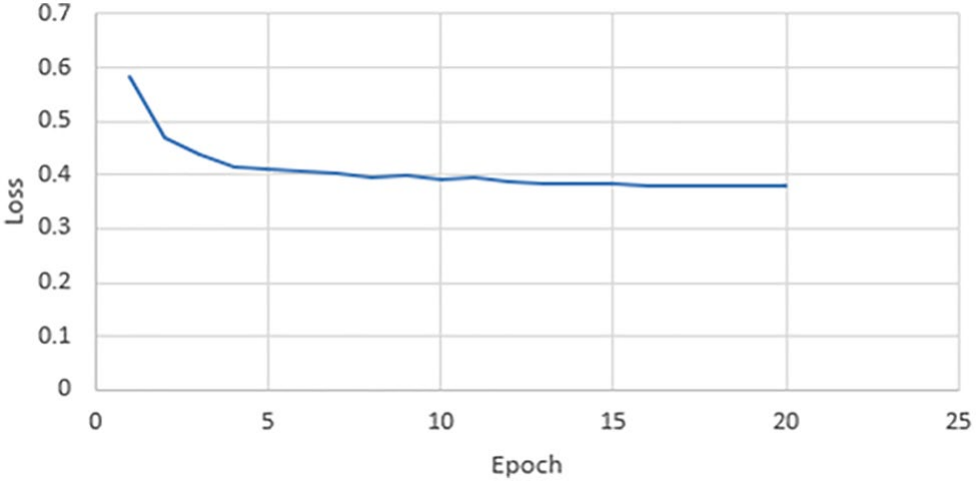
Validation loss of our model for VGG19 architecture.

**Figure 9:**
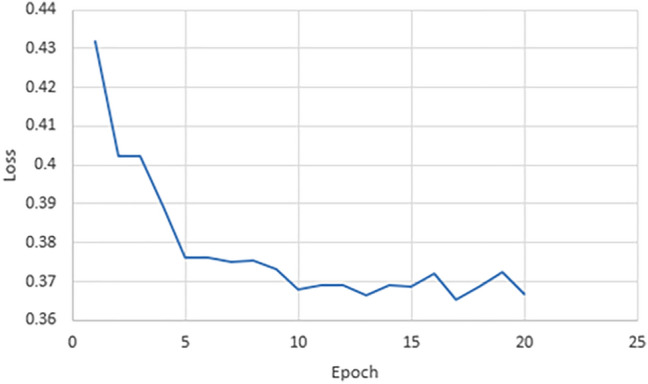
Validation loss of our model for VGG16 architecture.

Regarding loss, our model has achieved a very efficient results in the different models that we have used in our transfer learning technique, as shown in Figs. 7, 8 and 9. This show that our technique makes a very well learning process and it can handle the dataset to find efficient features that enable our model to identify the “Egyptian Cobra” bite in an effective manner.

### Time decision

Our target is to determine the species of the attacking snake which is the first and most important step to save the victim. So, this task should be performed as quick as possible. We compare our approach with previous works about time factor. The decision time depends on two factors: transmission time and prediction time. Regarding transmission time, our approach applies edge node paradigm where the classifier is installed in the edge points which are very close to the user. This causes less transmission time. Regarding prediction time, as shown in Table 6, our approach achieves less prediction time because of its easy implementation.

## Conclusion and future works

Egyptian Cobra is the most poisonous snake species in “Egypt”. It is able to kill a man in only 15 min. Whereas snake species identification is the first process to save a victim of snake bite, in this paper, we develop a model that can identify the bites of Egyptian Cobra with 91% and 96.7% of accuracy and sensitivity, respectively. We are the first to train our model on bite marks dataset compared to previous works that require the image of the attacking snake which is not practical at this accident. We also use cloud and edge computing to increase the availability and reduce the delay of the system. We also propose a novel technique in the cloud and edge architecture which is the re-training of the model with the wrong predictions, whereas we achieve a very efficient accuracy (91%) and is ready for its task, as we discussed above. However, we want to reach the best possible accuracy. So, the cloud architecture is responsible for training the model, after that the cloud is responsible for storing the updated dataset with any case that is predicted incorrectly by the model to retrain the model with this updated dataset to reach the best accuracy along time. So, the cloud is represented as central point to receive the updates of the dataset from all edge nodes to enhance model accuracy. Also, we use quantum PSO optimization algorithm to determine the optimal number of deployed edge nodes.

In spite of our manuscript handles this vital problem and proposes an efficient solution considering accuracy, cost, delay and other performance parameters, our proposal stills having some pitfalls whereas the dataset is so limited that is not enough to achieve efficient generalization. Also, the model is developed only for Egyptian Cobra bites, however there are also other some dangerous snake’s species that the model has not been trained on their bites. Also, we tried to handle the hardware requirements that the proposal needs using QPSO model to predict the optimal required number of edge nodes, but we intend to use more efficient and recent optimization algorithm to reach the best performance. Also, the deep learning technique is considered as a black box whereas it does not justify its predictions. So, we do not know why the model really outputs this classification, even if the result is accurate. In our future works, we intend to use the recent version of deep learning that is explainable deep learning that justifies the result of the model and clears the interested area of the model in the data samples that the model depends on them in its predictions. Therefore, we will try to handle all these drawbacks in our future works^27,28^.

## Data Availability

The datasets generated and analyzed during the current study are not publicly available due to the confidentiality of the patients, but are available from the corresponding author on reasonable request.
